# COVID-19 and prices of pulses in Major markets of India: Impact of nationwide lockdown

**DOI:** 10.1371/journal.pone.0272999

**Published:** 2022-08-25

**Authors:** Ranjit Kumar Paul, Md Yeasin

**Affiliations:** ICAR-Indian Agricultural Statistics Research Institute, New Delhi, India; Universiti Malaysia Sabah, MALAYSIA

## Abstract

The COVID-19 pandemic has impacted almost all the sectors including agriculture in the country. The present paper investigates the impact of COVID-19 induced lockdown on both wholesale and retail prices of major pulses in India. The daily wholesale and retail price data on five major pulses namely Lentil, Moong, Arhar, Urad and Gram are collected for five major markets in India namely Delhi, Mumbai, Kolkata, Chennai and Hyderabad during the period January, 2019 to September, 2020 from Ministry of Consumer Affairs, Food & Public Distribution, Government of India. The Government of India declared nationwide lockdown since March, 24, to May, 31, 2020 in different phases in order to restrict the spread of the infection due to COVID-19. To see the impact of lockdown on price and price volatility, time series model namely Autoregressive integrated moving average (ARIMA) model with error following Generalized autoregressive conditional heteroscedastic (GARCH) model incorporating exogenous variable as lockdown dummy in both mean as well variance equations. It is observed that in almost all the markets, lockdown has significant impact on price of the pulses whereas in few cases, it has significant impact on price volatility.

## 1. Introduction

The outbreak of covid-19 caused a disruptive damage to India and other nations in the world. In January 2020, The World Health Organization (WHO) declared COVID-19 as a pandemic disease. International Monetary Fund (IMF) predicted that the global economy is going to downsize by more than 3% in 2020. According to International Labour Organisation (ILO) the COVID-19 pandemic, along with lockdowns, mobility limitations and social-distancing norms has not only surge unemployment and income loss but also effected price inflation and spending pattern of the consumers. India, one of the most populated country, imposed a complete lockdown from 24^th^ March 2020. Unfortunately, lockdown was enforced shortly before the Rabi (Winter) season which is crucial time to harvest some of the major crops. Although the government of India relaxed the lockdown norms for agriculture related operations, still it affected demand and supply chains of agriculture commodities due to restrictions in transportation and labour mobility [1]. Consumers all across the world have reacted to the crisis and has caused to typical shopping behaviour. India leads the way, with 69–78% of customers expressing a desire to modify their purchasing habits in lockdown periods [2]. The lockdown resulted in a 23.9 percent drop in GDP in the first quarter (April to June) of 2020–21. Only the agriculture sector had positive growth (3.4 percent), although it is lesser than its previous quarter growth (5.9 percent). So, due to COVID-19 pandemic, Indian economy witnessed decline of agriculture growth by 2.5% [3]. Price fluctuations in agricultural commodities has a significant impact on poverty reduction and food security across the country. The supply of agricultural goods was particularly impacted, posing a major threat to the food and nutritional security of the most vulnerable section of society [4]. Bairagi et al. reported that price of basic food items such as wheat flour and rice in India increased significantly during the pandemic period [5]. Ruan et al. studied the impact of COVID-19 and nationwide lockdowns on vegetable prices in China [6]. They reported that the lockdown policy caused a large and immediate surge in price and price dispersion of Chinese cabbage. Akter examined the impact of ‘stay-at-home’ restrictions during of COVID-19 on food prices in 31 European countries [7]. It was found that the most significant surges in prices were meat, fish & seafood, and vegetables. Hobbs J assessed the implications of the COVID-19 pandemic for food supply chains in US-Canada border [8]. Yu et al. studied the impact of COVID-19 on food prices in China and reported no significant impact on rice and wheat flour prices, but significantly positive impact on cabbages prices [9]. Laborde et al. reported that COVID-19-related lockdown measures explain most of the fall in output, whereas declines in savings soften the adverse impacts on food consumption [10]. Ahmed et al. investigated the Impact of COVID-19 on agricultural production and distribution in South Asia and found that Livestock, vegetable, fruit, and fishing sector were more affected than the crop sector due to the pandemic [11]. Vyas et al. assessed the early impacts of the COVID-19 pandemic on the food supply chain and farm distress in India [12]. They showed wide-scale impacts across different components of the food supply chain. Kumari et al. reported that buyer and supplier uncertainty created by the COVID-19 lockdowns encouraged stakeholders to engage in relational governance initiatives [13]. Alina et al. studied the Impact of Covid-19 on the agri-food sector in Romania [14]. Janssen et al. found that it was more difficult for people to eat healthily during a COVID-19 confinement in terms of fresh fruit and vegetables [15]. The lockdown’s impact on the Indian agricultural sectors has been complicated and diversified across several sectors. The impact of the lockout on perishable food (milk, vegetables and fruits) products was worrisome, but there was also a substantial impact on the food grains, pulses, and oilseeds industry. Paul and Birthal investigated the impact of lockdown on wholesale and retail prices of tomato, onion and potato in major markets of India [16]. They reported surge in price volatility in all the studied commodities.

India is the world’s leading producer (25% of the world production), consumer (27% of the world consumption), and importer (14% of world imports) of pulses [17]. Approximately 20% area of total food grains cultivation is under the pulse’s cultivation and pulses accounts for 7% of the total food grains production in India [18]. Gram is the most dominant pulse with a share of around 40% in the total production followed by Arhar at 15 to 20% and Urad and Moong at around 8–10% each. Pulses are one of the important sources of protein in Indian diets. Depending on the species and variety, pulses have a protein content ranging between 17 to 32 percent. Pulses contain good amount of protein, carbohydrates, and dietary fibre. A good description of nutritional values of different pulses is given by Venkidasamy et al [19]. Urad is a rich source of protein, fat, carbohydrates, and Vitamin B. Fiber, protein, and folic acid are all abundant in arhar dal. Gram contains high-protein, zinc, and calcium. Lentil is a high-protein, vitamin B_1_, potassium, and amino acid-rich pulse. In Moong, Phosphorus, magnesium, potassium, and other essential nutrients may be found. Therefore, one pulse may substitute other with respect to nutritional value.

Price of pulses in retail as well as wholesale markets has been fluctuated during and after lockdown due to disrupt supply chains and change in consuming pattern of consumers. A significant change in the consumption habits of consumers was observed during the lockdown phase and the consumers were spending mostly on essentials commodities. In this study we investigated the effect of lockdown on the prices of major pulses in India’s leading wholesales and retails markets. These issues are more prominent in the context of a developing country where the food supply chain is long and fragile [20]. Generally, pulses exhibit high price volatility [21]. Pulse price volatility is one of the key concerns for policymakers. Volatility is the sudden unexpected rise or fall in the series. A large number of models have been developed to capture volatility since development of Autoregressive conditional heteroscedastic (ARCH) model by Engle (1982) followed by Generalized ARCH (GARCH) model by Bollerslev (1986) [22,23]. There are various ways of measuring price volatility. Among them, GARCH model is most popular model due to its wide applicability into various field [24]. Various researchers already proved supremacy of GARCH model in volatility estimation in agriculture and allied sectors [25]. Paul *et al*. demonstrated that GARCH model is superior than usual ARIMA model for forecasting the spices export from India [26]. Ghosh *et al*. reported efficiency of nonlinear time series model e.g. GARCH in modelling conditional volatility; Paul *et al*. studied application of ARIMAX-GARCH model for forecasting monsoon rainfall in India [27,28]. Paul applied GARCH model by introducing exogenous variable in mean model for forecasting wheat yield [29]. Rakhsit *et al*. demonstrated the different extension of GARCH model to capture the asymmetricity present in onion price volatility [30]. When volatility is influenced by exogeneous variables, the simple GARCH model is not sufficient to model volatility. GARCH with exogenous variables (GARCH-X) has the potential to capture the volatility within the study series and also evaluate the influence of environmental influences [31]. In order to improve the forecast accuracy of GARCH model, the external demand influencing factors (X) needs to be incorporated in the forecasting model. If these external factors are excluded, the GARCH model may encounter biased estimates of volatility. Various researchers have already studied GARCH model with covariates; such as Brenner *et al*. and Patton used interest rate levels as exogeneous variables; Apergis used certain macroeconomic fundamentals as covariates to estimate stock prices; Yeasin et al. modelled domestic price index by using two external variables [31–34].

In the present investigation, we formulated our study to analyse the volatility of wholesale and retail prices of major pulses in account for the influence of lockdown. We have considered five pulses namely gram, arhar, urad, moong and lentils in five metro cities, i.e. Delhi, Mumbai, Kolkata, Hyderabad and Chennai. The primary objective of this research is to assess the impact of lockdown restrictions on prices of pulses in the Indian market. The exogenous variable may affect both the mean and variance model of GARCH collectively. To understand effect of lockdown on prices of pulses, we employed GARCH-X covariate (lockdown dummy) in both the mean and variance equations of GARCH model. The paper is organized as follows: Section 2 describes the methodology involved in this study. Section 3 deals with the results and discussion. Section 4 draws conclusion.

## 2. Methodology

The Box-Jenkin’s Autoregressive Integrative Moving Average (ARIMA) model gained enormous popularity in class of linear time series model. But when the series is nonlinear and the conditional variance of error term is heteroscedastic in nature, the linear ARIMA model fails to describe behavior of study variable. An Autoregressive Conditional Heteroscedastic-Lagrange Multiplier (ARCH-LM) test is used to detect the nonlinearity and conditional heteroscedasticity in ARIMA residuals [22]. The ARCH-LM test is conducted as follows:

Assuming that *ε*_*t*_ be the residual series, the Lagrange Multiplier (LM) test for $\varepsilon_{t}^{2}$ is used to check for presence of conditional heteroscedasticity. The null hypothesis of the test is specified as H_0_: *a*_*i*_ = 0, i = 1, 2, …, q in the following regression equation:

$$\varepsilon_{t}^{2}=a_{0}+a_{1}\varepsilon_{t-1}^{2}+\cdots +a_{q}\varepsilon_{t-q}^{2}+e_{t};\;t=(q+1)\ldots T$$ (1)

where e_*t*_ denotes the error term, q is the positive integer, and T is the number of observations.

The test statistics is

$$F=\frac{({SSR}_{0}-{SSR}_{1})/q}{{SSR}_{1}/(T-q-1)}$$ (2)

where SSR_0_ = $\sum_{t=q+1}^{T}{\left(\varepsilon_{t-}^{2}\bar{\omega }\right)}^{2}$, $\bar{\omega }=\sum_{t=q+1}^{T}\varepsilon_{t}^{2}/T$ is sample mean of {$\varepsilon_{t}^{2}$}, and SSR_1_ = $\sum_{t=q+1}^{T}{eˆ}_{t}^{2}$, *e*_*t*_ is the least square residual.

The test statistic F asymptotically follows Chi square distribution with q degrees of freedom.

The presence of volatility makes it challenging for linear ARIMA model to adequately describe the study variable. If ARCH-LM test indicates the presence of ARCH effect, an appropriate nonlinear time series model may be implemented to capture the complex behavior of data. Generalized autoregressive conditional heteroscedastic (GARCH) model is one of popular models in the class of nonlinear time series models [22]. To include the effect of exogenous variable, an improvement of GARCH model, GARCH-X model, is developed. A brief description of the GARCH and GARCH-X models along with the estimation procedures are given below:

### GARCH model

GARCH model is a weighted average of past squared residuals and also includes declining weights for the residuals that never reach zero. GARCH process also models the lagged conditional variances. If *y*_*t*_, *y*_*t*−1_, *y*_*t*−2_,…, *y*_2_, *y*_1_ are the observation of a volatile time series data, then GARCH (p, q) model is defined as

$$\in_{t}=\sqrt{{\sigma^{2}}_{t}}.\varepsilon_{t}{;\;\in_{t}/\varphi }_{t-1}∼N(0,{\sigma^{2}}_{t}),$$ (3)

$${\sigma^{2}}_{t}=\omega +\sum_{i=1}^{p}\alpha_{i}{\in^{2}}_{t-i}+\sum_{j=1}^{q}\beta_{j}{\sigma^{2}}_{t-j}$$

where *μ*>0, *α*_*i*_≥0, *i* = 1,2,…,*q* for all i and *β*_*j*_≥0, *j* = 1,2,…,*p* and $\sum_{i=1}^{q}\alpha_{i}+\sum_{i=1}^{q}\beta_{j}<1.$ *ε* is distributed i.i.d. with zero mean and unit variance. We can add *η*_*t*_ = ∈^2^_*t*_−*σ*^2^_*t*_ to both side of the equation to express the model in the form of ARMA (mean model) model.


$${\in^{2}}_{t}=a_{0}+\sum_{i=1}^{max(p,q)}\left(\alpha_{i}+\beta_{j}\right){\in^{2}}_{t-i}+\sum_{j=1}^{q}\beta_{j}\eta_{t-j}$$ (4)

Mean equation (ARIMA) of the GARCH model can be represented by the following equation

$$\varphi (B){\left(1-B\right)}^{d}Y_{t}=\theta (B)\varepsilon_{t}$$ (5)

where, *φ*(*B*) = 1−*φ*_1_*B*−*φ*_2_*B*^2^−⋯−*φ*_*p*_*B*^*p*^ = the autoregressive operator of order p;

*φ*_1_, *φ*_2_,…,*φ*_*p*_ = the corresponding autoregressive parameters; *θ*(*B*) = 1−*θ*_1_*B*−*θ*_2_*B*^2^−⋯−*θ*_*q*_*B*^*q*^ = the moving average operator of order q; *θ*_1_, *θ*_2_,…,*θ*_*q*_ = the associated non-seasonal moving average parameters; (1−*B*)^*d*^ = the differencing operator of order d to produce stationarity of the d^th^ differenced data. In Eq (3) B is used as a backshift operator on *Y*_*t*_ and is defined as *B*^*i*^(*Y*_*t*_) = *B*^*i*^(*Y*_*t*−1_).

### GARCH-X model

In GARCH-X model, the effect of external covariates is taken into consideration. This improvement of GARCH model has the ability to capture the real situation more efficiently. The algorithms of the GARCH-X model can be summarised into four steps:

- **Model identification:** In this step, we select the appropriate GARCH (p, q) order. Tsay (1987) stated that GARCH (1, 1) model provides the best fit than its higher order as higher order of GARCH model involved greater complication and also yield no significant enhancements in goodness of fit. So, we used GARCH (1, 1) as predecessor model of the GARCH-X formulations [35].
- **Inclusion of exogenous variables:** This step is regarded as one of the most essential steps in GARCH-X procedure. Here, we decide how many and what kinds of exogenous variables can be included in the model. There are no particular rules for determining the suitable numbers and kind of exogenous variables. This solely depends on the objective of the study. In this study we incorporate a covariate *viz*. lockdown as a dummy. For lockdown period we define lockdown dummy as:
X=1,iflockdownispresent
=0,otherwise
  Now the GARCH-X model is developed by including the covariate in both the mean and variance equation of GARCH model.
$$Mean\;model:\;\varphi (B){\left(1-B\right)}^{d}Y_{t}=\theta (B)\varepsilon_{t}+\gamma_{1}X_{t}$$ (6)
$$Variance\;model:\;{\sigma^{2}}_{t}=\omega +\sum_{i=1}^{p}\alpha_{i}{\in^{2}}_{t-1}+\sum_{j=1}^{q}\beta_{j}{\sigma^{2}}_{t-1}+\gamma_{2}X_{t}$$ (7)
  Similar to the GARCH, conditions for stationary of the GARCH-X model are *μ*>0, *α*_*i*_≥0 for all i; *β*_*j*_≥0 and $\sum_{i=1}^{q}\alpha_{i}+\sum_{i=1}^{q}\beta_{j}<1.$ The parameter *γ*_1_ and *γ*_2_ represent the impact of the covariates on the mean and conditional variance of series respectively.
- **Parameters estimation:** Similar to the GARCH model, parameters of the GARCH-X model are estimated by Quasi-maximum likelihood estimation (QMLE) technique.
- **Diagnosis the fitness of the model:** In this final step of the model, we diagnosed GARCH-X model with various selection criteria (Akaike, Bayes, Shibata and Hannan-Quinn information criteria) and residuals testing. Residual diagnostic is an important tool validate a model. The assumptions of the error in the model can be tested using residual analysis. Ljung-Box test have been performed to check adequacy of the fitted GARCH and GARCH-X model [36]. To test the structural breaks and ARCH effect in residuals, we have implemented Nyblom Stability and ARCH-LM test respectively [22,37]. All the above tests ensure that the models build up for the data under study are correctly specified and adequate.

## 3. Result and discussions

### Data description

**Fig 1:**
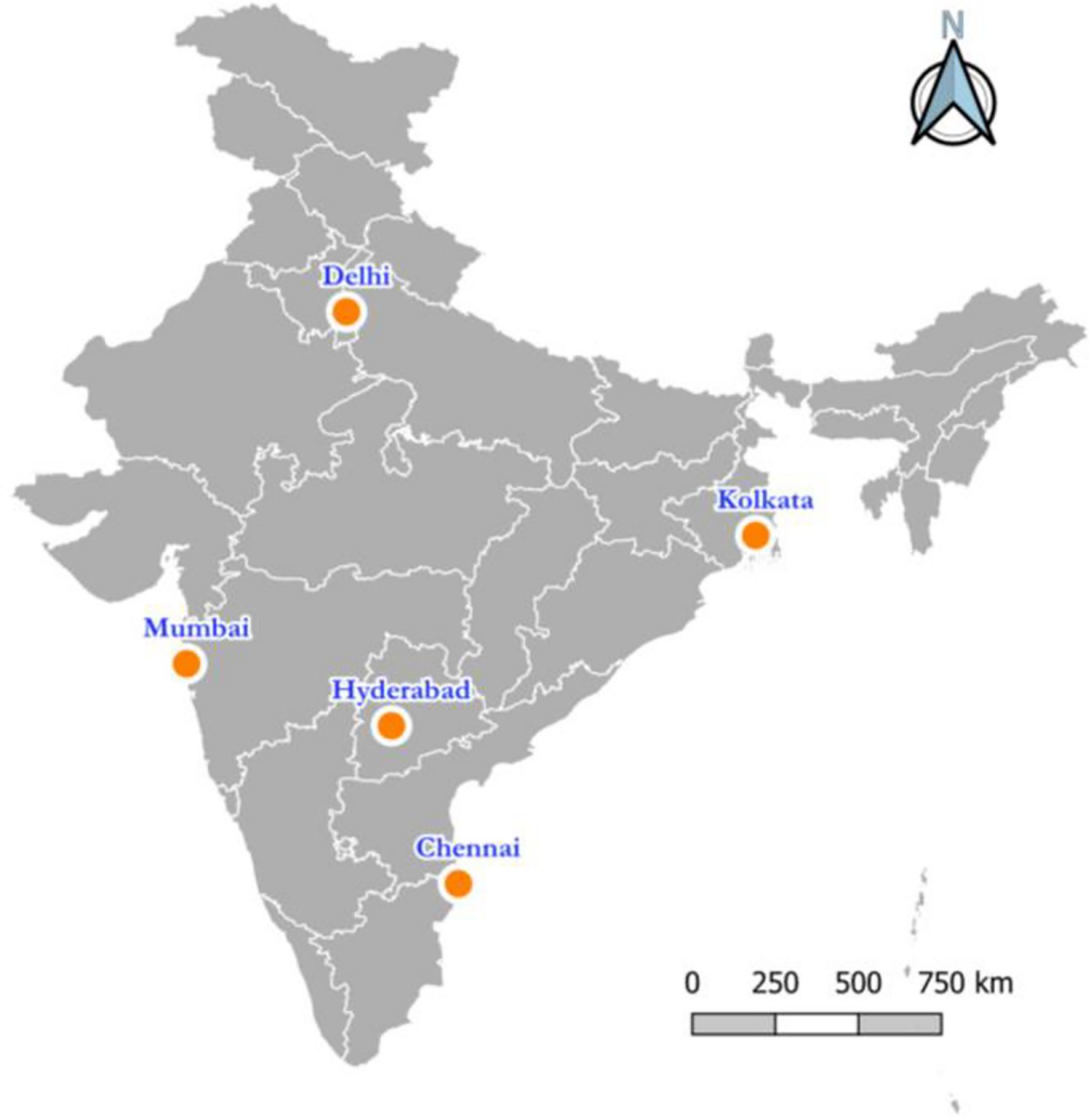
The study locations.

Daily wholesale and retail price data during the period 1^st^ January, 2019 to 30^th^ September, 2020 for five major pulses (gram, arhar, urad, moong and lentils) in five metro cities of India namely Delhi, Mumbai, Kolkata, Chennai and Hyderabad have been collected from Department of Consumer Affairs, Ministry of Consumer Affairs, Food & Public Distribution, Government of India. The study locations are displayed in Fig 1. India implemented four nationwide lockdowns consecutively, beginning from March 25, 2020 to May 31, 2020. The first lockdown was of 21 days duration (March 25–April 14); the second of 19 days (April 15–May 3); the third of 14 days (May 4–17), and the fourth of14 days (May 18–31).

**Fig 2:**
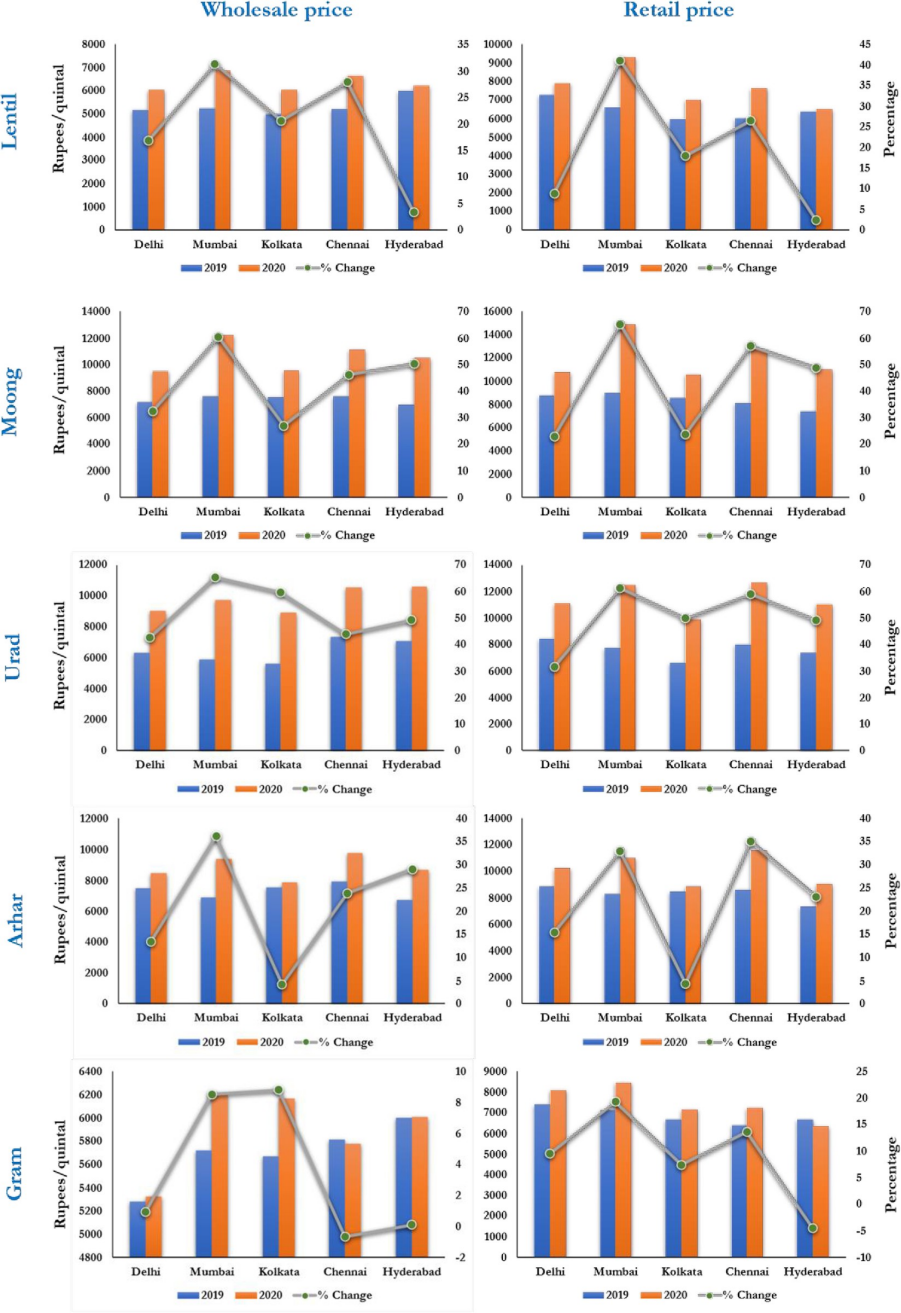
Average price and % change in price of pulses in different markets of India. Note: % change indicates the percentage change in prices during lockdown period of 2020 compared to the same period in 2019.

Fig 2 presents the average wholesale and retail prices of different pulses in different markets in India during the entire lockdown period (March 25-May 31, 2020), the corresponding periods of 2019 and also the full sample period i.e. during January, 2019 to September, 2020. A perusal of Fig 2 indicates that the prices (both retail and wholesale) of all the five pulses in all the studied markets increased in 2020 during the lockdown period as compared to previous year. Also, when considering full sample of data, the wholesale and retail prices of Lentil, Moong and Gram are maximum in Mumbai, whereas Chennai experienced highest prices of Urad and Arhar.

Moreover, percentage changes in average prices of pulses in all the studied markets during lockdown period i.e. March-May, 2020 with the same period in 2019 have been computed and displayed in Fig 2. Overall, Mumbai has the greatest increase in retail and wholesale prices, followed by Chennai. Delhi had the lowest price increases for both retail and wholesale, followed by Kolkata. In terms of pulses, gram is the least affected, followed by lentil while urad is the most affected, followed by moong. When we examine the change in price for wholesale and retail prices of pulses, we have seen a mixed picture. In the Hyderabad and Kolkata markets, changes in retail price are less than changes in wholesale price for all five pulses, but in Chennai, changes in retail price are more than changes in wholesale price for four out of five pulses. Mumbai and Delhi displayed a varied pattern.

**Fig 3:**
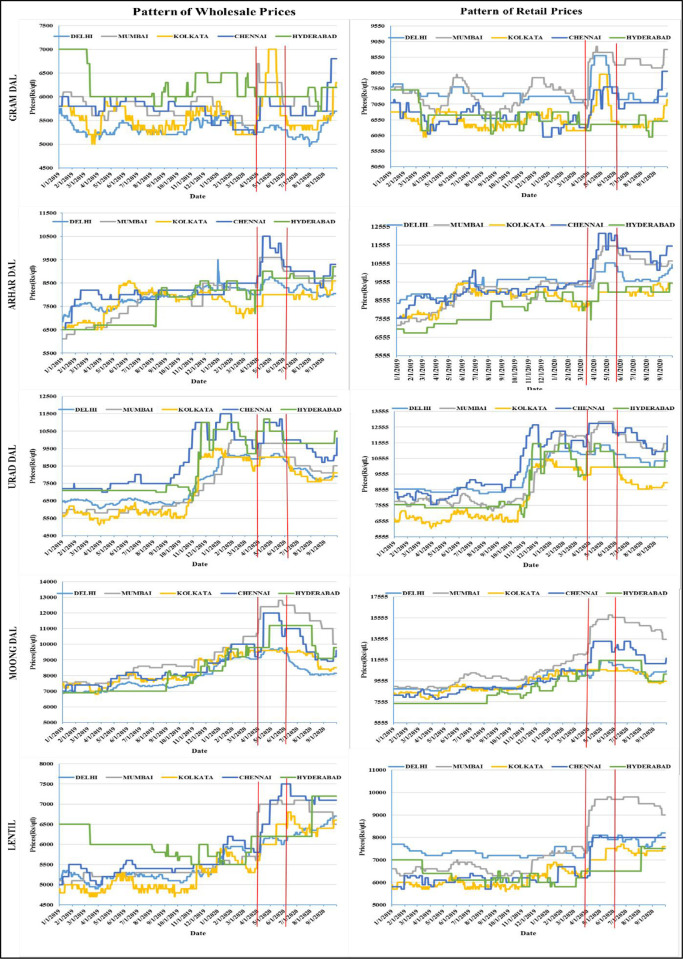
Pattern of wholesale and retail prices of pulses.

The price behaviour during the studied period can also be seen in Fig 3. The red coloured vertical lines indicate the duration of lockdown. Looking at the price trend, it was evident that prices of pulses in both wholesale and retail began to rise in chosen five Indian markets following the implementation of the lockdown. In the instance of Gram, the wholesale price volatility was highest in Kolkata, whereas the retail price variation was highest in Mumbai and lowest in Hyderabad. The Kolkata market showed stable price, whereas the Chennai market fluctuated the most in both retail and wholesale Arhar prices. For Urad wholesale prices, the largest volatility was noted in the Chennai and Hyderabad markets, and the lowest in Kolkata and Delhi, but for retail prices, the highest fluctuation was recorded in Chennai and Mumbai, and Kolkata had consistent price. The wholesale and retail prices of Moong varied greatly in the Mumbai and Kolkata markets. For Lentil, wholesale prices, the largest variance was seen in the Chennai market and the lowest variation was recorded in the Delhi market, but for lentil retail prices, the highest fluctuation was observed in Mumbai and lowest in Hyderabad. It is to be noted that higher price variation has been observed in retail markets as compared to the wholesale markets for all the pulses.

### Application of GARCH-X model

**Fig 4:**
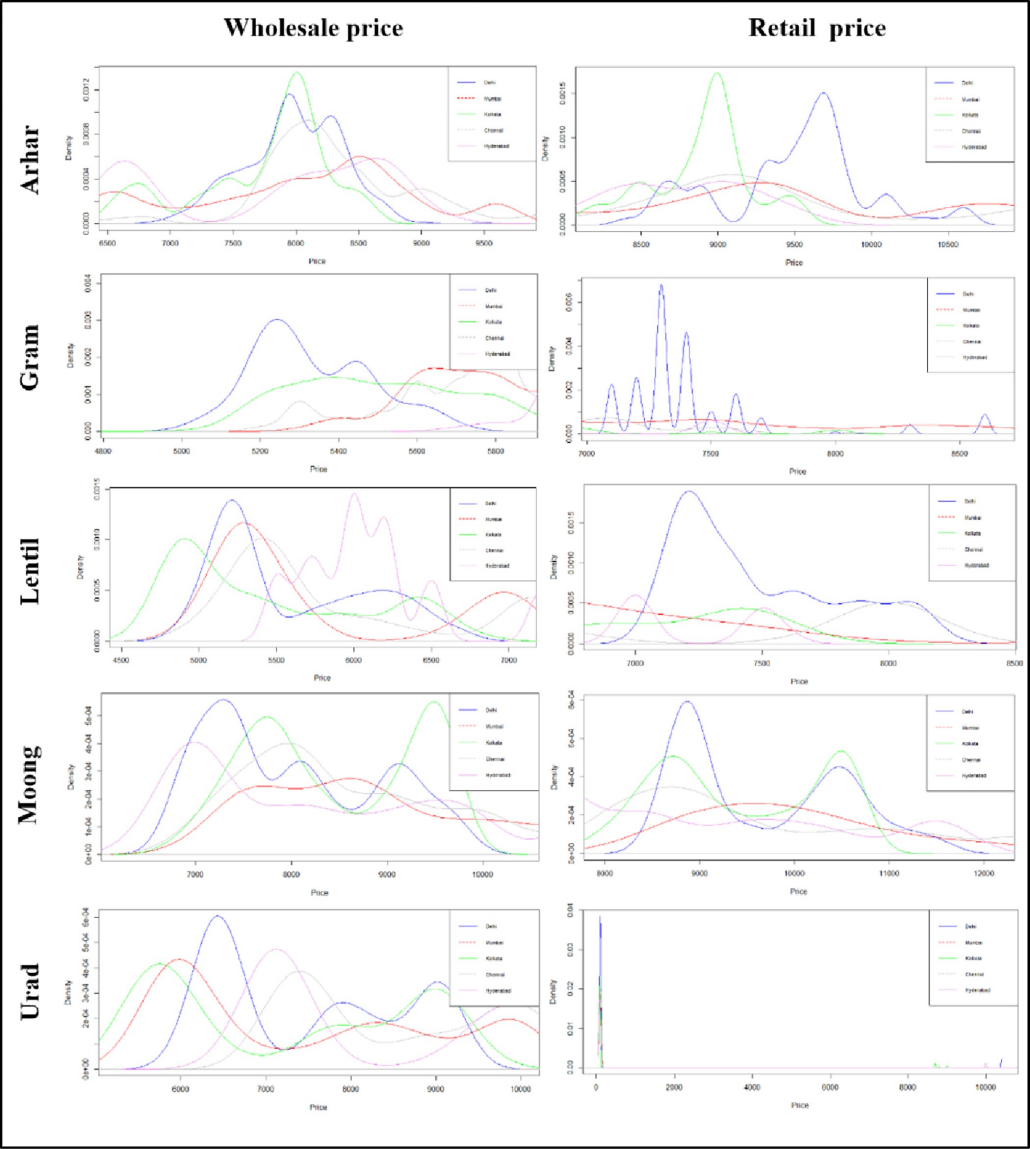
Kernel density estimate of wholesale and retail prices of pulses in different markets.

From the above discussion, it is clear that price fluctuations in the both markets are not only caused by usual inflation in the market, but also by the influence of the lockdown restrictions. The estimate of kernel density of the wholesale and retail prices of pulses in different markets is reported in Fig 4. A perusal of Fig 4 confirms the significant departure from normality of all the price series. Therefore, underlying error distribution is considered to be generalized error distribution as the data does not follow normality. To assess the statistical impact of the lockdown, we model the daily return price of pulses using an ARIMA-GARCH-X model with the lockdown dummy as a covariate. First, the ARIMA models have been implemented and residuals of the models were tested using ARCH-LM test. The test results evidenced the presence of ARCH effect in data sets. Hence, the GARCH-X model is more suitable to evaluate the impact of the lockdown on pulse prices. To check the model adequacy Ljung-Box, ARCH-LM and Nyblom Stability test have been implemented and test results showed that all the GARCH-X models have been correctly specified and the residuals of the models fulfilled all its assumptions. The parameter estimates of the fitted models are reported in Tables 1–5 for different pulses. The parameters μ, *φ*_1_, *θ*_1_, *γ*_1_, *ω*, *α*_1_, *β*_1_, *γ*_2_ respectively denotes the constant in mean model, AR coefficient, MA coefficient, impact of lockdown on mean price return, constant of variance equation, ARCH effect, GARCH effect and impact of lockdown on price volatility.

**Table 1:** Parameters estimate of GARCH-X model for gram.

Parameters	Wholesale price					Retail price				
	Delhi	Mumbai	Kolkata	Chennai	Hyderabad	Delhi	Mumbai	Kolkata	Chennai	Hyderabad
**μ**	0.0001	-0.0006 ***	0.0000	-0.0002	-0.0023 ***	-0.0001 ***	0.0001	0.0000	0.0003 ***	-0.0006 ***
***φ***_**1**_	-0.0833	-0.0187 ***	-0.3541	0.6796	0.2595	-0.0737 ***	0.9426 ***	0.4471 **	-0.3401 ***	0.9052 ***
***θ***_**1**_	0.0486	-0.0195 ***	0.2616	-0.6889	-0.5891 ***	0.0584 ***	-0.9104 ***	-0.5635 ***	0.2943 ***	-0.9927 ***
***γ***_**1**_	-0.0004	-0.0035 ***	0.0017	0.0033 **	0.0023 ***	0.1012 ***	0.0015	0.0004	0.0095 ***	0.0012 ***
***ω***	0.0000 ***	0.0000	0.0000	0.0000 ***	0.0000 *	0.0000	0.0000 ***	0.0001 ***	0.0000	0.0000
***α***_**1**_	0.1215**	0.0534 ***	0.1024 **	0.1240*	0.2600 **	0.0501 *	0.1650**	0.1570**	0.0549 *	0.1892 **
***β***_**1**_	0.8743 ***	0.9251 ***	0.7000 ***	0.7909 ***	0.6774 ***	0.9007 ***	0.4250**	0.3421**	0.8185 ***	0.6534 ***
***γ***_**2**_	0.0000	0.0000	0.0000	0.0000 ***	0.0000	0.0000	0.0005 ***	0.0003 ***	0.0000	0.0000

Note

***, **, * denotes significant at 1%, 5% and 10% level of significate respectively.

**Table 2:** Parameters estimate of GARCH-X model for arhar.

Parameters	Wholesale price					Retail price				
	Delhi	Mumbai	Kolkata	Chennai	Hyderabad	Delhi	Mumbai	Kolkata	Chennai	Hyderabad
**μ**	0.0001	0.0008 *	-0.0003	0.0002	0.0003 ***	0.0000 ***	0.0005	0.0005	0.0005	-0.0002 ***
***φ***_**1**_	0.9522 ***	0.6162	0.1187	-0.1994	-0.4673 **	-0.3152	0.0770	0.4669	-0.0146	0.8657 ***
***θ***_**1**_	-0.8621 ***	-0.5880	-0.1603	0.2064	0.6029 ***	0.4733 ***	-0.0753	-0.4671	0.0145	-0.9816 ***
***γ***_**1**_	0.0006	-0.0020	0.0012	0.0007	-0.0005	0.0002	0.0003	0.0004	0.0006	-0.0010 ***
***ω***	0.0000 ***	0.0000	0.0000 ***	0.0000 ***	0.0000 ***	0.0000 ***	0.0001 ***	0.0002 ***	0.0001 ***	0.0000
***α***_**1**_	0.0770 ***	0.103 *	0.1244 **	0.1091 ***	0.1293 **	0.4414 ***	0.0482 *	0.0146	0.1250*	0.2057 ***
***β***_**1**_	0.8737 ***	0.7990 ***	0.8613 ***	0.8826 ***	0.8112 ***	0.3571 ***	0.1200**	0.1828**	0.4747 **	0.6810 ***
**γ** _**2**_	0.0000	0.0000	0.0000	0.0000	0.0000 **	0.00002 ***	0.0003 ***	0.0000	0.0000	0.0000

Note

***, **, * denotes significant at 1%, 5% and 10% level of significate respectively.

**Table 3:** Parameters estimate of GARCH-X model for urad.

Parameters	Wholesale price					Retail price				
	Delhi	Mumbai	Kolkata	Chennai	Hyderabad	Delhi	Mumbai	Kolkata	Chennai	Hyderabad
**μ**	0.0002	0.0025 ***	0.0002	0.0004 **	0.0008 ***	0.0004	0.0003	0.0003	0.0005 ***	0.0033 ***
***φ***_**1**_	-0.4484 ***	0.0043 ***	0.6502 *	0.9405 ***	-0.1703 ***	0.9551 ***	-0.0018	0.4313 *	0.9944 ***	0.0546 ***
***θ***_**1**_	0.4308 ***	0.0050 ***	-0.7161 **	-0.9356 ***	--	-0.9314 ***	--	-0.5411 ***	-1.0000 ***	-0.2289 ***
***γ***_**1**_	0.0045 ***	1.4088 ***	0.0011 **	0.0038 *	-0.0084 ***	0.0012	0.0015	0.0007	0.0048 **	0.1869 ***
***ω***	0.0000	0.0000	0.0000 ***	0.0000 **	0.0000	0.0000 ***	0.0000 ***	0.0000 ***	0.0000	0.0000
***α***_**1**_	0.1571 ***	0.1501 ***	0.0126	0.1200*	0.0500	0.0212	0.0777 *	0.1003*	0.1000*	0.0502 *
***β***_**1**_	0.8094 ***	0.8002 ***	0.9635 ***	0.7853 ***	0.9002 ***	0.9346 ***	0.5704 ***	0.8947 ***	0.7963 ***	0.9014 ***
***γ***_**2**_	0.0000	0.0000	0.0000	0.0000 ***	0.0000	0.0000 ***	0.0001 ***	0.0000	0.0000 ***	0.0000

Note

***, **, * denotes significant at 1%, 5% and 10% level of significate respectively.

**Table 4:** Parameters estimate of GARCH-X model for moong.

Parameters	Wholesale price					Retail price				
	Delhi	Mumbai	Kolkata	Chennai	Hyderabad	Delhi	Mumbai	Kolkata	Chennai	Hyderabad
**μ**	0.0004	0.0003	0.0002	0.0015 ***	0.0003 ***	0.0014 ***	0.0002	0.0002	0.0004 ***	0.0000
***φ***_**1**_	0.9801 ***	-0.1218	-0.1291	0.0059	--	-0.5784 ***	0.9861 ***	0.0706	1.0000 ***	-0.0280
***θ***_**1**_	-0.9290 ***	0.1209	0.1004	0.0059	--	0.5465 ***	-0.9684 ***	-0.0799	-1.0000 ***	-0.0308
***γ***_**1**_	0.0019 *	0.0025 **	-0.0001	0.0221 ***	-0.0003 ***	0.0108 ***	0.0039 *	-0.0002	0.0054 **	0.0038 ***
***ω***	0.0000 ***	0.0000	0.0000 ***	0.0000	0.0000	0.0000	0.0000 ***	0.0000	0.0000 ***	0.0000
***α***_**1**_	0.1311 **	0.1014 **	0.1201 **	0.1509 **	0.0834 *	0.0503 *	0.1020*	0.1014 **	0.1015*	0.1216 **
***β***_**1**_	0.8041 ***	0.7997 ***	0.7903 ***	0.8051 ***	0.8208 ***	0.9023 ***	0.6350***	0.7968 ***	0.7639 ***	0.8262 ***
***γ***_**2**_	0.0000 ***	0.0000	0.0000	0.0000	0.0000	0.0000 ***	0.0003 ***	0.0000	0.0000 ***	0.0000

Note

***, **, * denotes significant at 1%, 5% and 10% level of significate respectively.

**Table 5:** Parameters estimate of GARCH-X model for lentil.

Parameters	Wholesale price					Retail price				
	Delhi	Mumbai	Kolkata	Chennai	Hyderabad	Delhi	Mumbai	Kolkata	Chennai	Hyderabad
**μ**	0.0003	0.0003	0.0002	0.0001	0.0004 ***	-0.0014 ***	0.0000	0.0002	0.0029 ***	0.0003 ***
***φ***_**1**_	-0.0018	0.1166	-0.0933 **	-0.0348	-0.0012	-0.0077	0.7287	0.5165 ***	-0.0323 ***	0.8759 ***
***θ***_**1**_	--	-0.1669 ***	--	--	--	--	-0.6889	-0.7168 ***	-0.0357 ***	-0.9162 ***
***γ***_**1**_	0.0015	0.0226 ***	0.0028 *	0.0095 ***	-0.0004 **	0.0039 ***	0.0041	0.0027 ***	-0.0007 ***	-0.0003 **
***ω***	0.0000 ***	0.0000	0.0000 ***	0.0000	0.0000	0.0000	0.0000 ***	0.0000	0.0000	0.0000
***α***_**1**_	0.0777 *	0.0537 *	0.1102 **	**	0.0516 *	0.0547 *	0.0212	0.128 **	0.0530 *	0.0504 *
***β***_**1**_	0.5704 ***	0.8615 ***	0.8341 ***	0.8100 ***	0.9074 ***	0.9212 ***	0.6540**	0.857 ***	0.9105 ***	0.9037 ***
***γ***_**2**_	0.0001 ***	0.0000	0.0000	0.0000	0.0000	0.0000	0.0004 ***	0.0000	0.0000	0.0000

Note

***, **, * denotes significant at 1%, 5% and 10% level of significate respectively.

A perusal of the parameters estimates reported above indicate that, in the case of gram wholesale price, effect of lockdown was significant in the mean model for Mumbai, Chennai, and Hyderabad markets and in the variance model for Chennai market, whereas in case of retail price, effect of lockdown was significant in the mean model for Chennai market and in the variance model for Mumbai and Kolkata markets (Table 1). In case of arhar wholesale price, significance in lockdown was seen only for the Hyderabad market in the variance model, but in case of retail price, significance was detected only for the Hyderabad market in the mean model and the Mumbai and Delhi markets in the variance model (Table 2). In case of urad wholesale price, lockdwon was significant for all five markets in the mean model and only for the Chennai market in the variance model, whereas in retail price, lockdown was significant for the Chennai and Hyderabad markets in the mean model and for the Delhi, Mumbai, and Chennai markets in the variance model (Table 3). In case of moong, significant effect of lockdown was found for the markets of Delhi, Mumbai, Chennai, and Hyderabad in the mean model and Delhi in the variance model, while in retail price, effect of lockdown was significant for the markets of Delhi, Mumbai, Chennai, and Hyderabad in the mean model and Delhi, Mumbai, and Chennai in the variance model (Table 4). In case of lentil wholesale price, lockdown effect was significant in the mean model for Mumbai, Kolkata, Chennai, and Hyderabad markets and in the variance model for Delhi market, whereas in the case of retail price, it was significant in the mean model for Delhi, Kolkata, Chennai, and Hyderabad markets and in the variance model for Mumbai market (Table 5). The total of the Alpha and Beta parameters for all instances was quite high (near to 1), indicating that volatility was well captured. Based on Tables 1–5, we can conclude that the effect of lockdown was observed in nearly all pulse prices, although arhar has a comparatively modest effect of lockdown in comparison to other pulses.

## 4. Conclusion

The Indian government implemented a nationwide lockdown starting March 24, 2020, with some exceptions for agricultural operations. The lockdown has little effect on pulse production, but it has an impact on the demand and supply chain owing to poor transit. COVID-19 has revealed weaknesses and imbalances in the Indian agricultural demand-supply system. In the present investigation, the impact of lockdown on wholesale and retail prices of pulses in major Indian markets have been evaluated. It revealed that, the lockdown has a significant impact on both the wholesale and retail prices of pulses. Among the studied markets, Mumbai has seen the highest increase in retail and wholesale prices, followed by Chennai during the lockdown period. Delhi had the lowest price increases for both retail and wholesale, followed by Kolkata. In case of pulses, due to lockdown, gram is the least impacted, followed by lentil, and urad is the most affected, followed by moong. For arhar, the lockdown has a comparatively modest effect. The imposition of lockdown not only impacted the price of pulses but also its volatility significantly. Multiple national lockdowns have resulted irregular flow of raw materials and finished goods, disrupting the supply chain. The inclusion of lockdown as exogenous variable in both mean and variance equation of fitted model could effectively capture the variation in prices across the different markets. In future study, the other variants of GARCH model including machine learning techniques may be applied to model the volatility in agricultural commodity prices.
